# Bridging the Gap: Awareness, Knowledge, and Challenges of Living with Celiac Disease in Bulgaria

**DOI:** 10.3390/nu17071267

**Published:** 2025-04-04

**Authors:** Rouzha Pancheva, Jernej Dolinsek, Marlena Panayotova, Ivan Yankov, Denitza Kofinova, Silviya Nikolova, Mila Baycheva, Miglena Georgieva

**Affiliations:** 1 Department of Hygiene and Epidemiology, Faculty of Public Health, Prof. Paraskev Stoyanov Medical University, 9000 Varna, Bulgaria; 2 Department of Pediatrics, University Medical Center Maribor, 2000 Maribor, Slovenia; jernej_dolinsek@hotmail.com; 3 Department of Pediatrics, Medical Faculty, University of Maribor, 2000 Maribor, Slovenia; 4 Department of Pediatrics, Faculty of Medicine, Trakia University, 6000 Stara Zagora, Bulgaria; marlenapanayotova@gmail.com; 5 Department of Pediatrics, Medical University of Plovdiv, 4000 Plovdiv, Bulgaria; epediatrics@abv.bg; 6 Department of Pediatric Surgery, University Multiprofile Hospital for Active Treatment and Emergency Medicine “N.I. Pirogov”, 1000 Sofia, Bulgaria; kofinova@abv.bg; 7 Department of Social Medicine and Healthcare Organization, Faculty of Public Health, Prof. Paraskev Stoyanov Medical University, 9000 Varna, Bulgaria; silviya.p.nikolova@gmail.com; 8 Department of Gastroenterology and Hepatology, University Children’s Hospital, Medical Univerity of Sofia, 1000 Sofia, Bulgaria; mila.baycheva@gmail.com; 9 Department of Pediatrics, Faculty of Medicine, Prof. Paraskev Stoyanov Medical University, 9000 Varna, Bulgaria; mgeorgieva7@yahoo.com

**Keywords:** coeliac, child, awareness, diagnosis, diet adherence

## Abstract

**Background:** Celiac disease (CD) is a chronic immune-mediated systemic disorder induced by gluten in genetically predisposed individuals, requiring lifelong management through a strict gluten-free diet (GFD). Although its global prevalence is around 1%, awareness and diagnosis remain suboptimal, contributing to challenges in disease management. **Objectives:** To assess the awareness, knowledge, and experiences of Bulgarian CD patients and caregivers regarding CD, diagnosis, and dietary adherence. **Methods:** A structured survey was conducted to evaluate patient and caregiver knowledge, awareness, and experiences with CD, focusing on the diagnostic process and dietary practices. Data were collected from a sample of Bulgarian CD patients and their caregivers. **Results:** The majority of the 191 respondents (94%) recognized CD as a lifelong condition, but only 26.7% correctly identified its autoimmune, systemic nature. The average diagnostic delay was 8.1 months, with over 50% of patients relying on serological tests alone, consistent with recent non-biopsy guidelines. Dietary adherence was significantly hindered by misconceptions about gluten-containing grains and societal barriers. Notably, 83.6% of participants reported bringing their own food when eating outside. **Conclusions:** The findings underscore the need for targeted public health initiatives, enhanced healthcare provider training, and improved dietary education to address knowledge gaps, expedite diagnosis, and improve dietary adherence. Such interventions could help reduce the psychosocial burden of CD and enhance the quality of life for affected individuals.

## 1. Introduction

Celiac disease (CD) is a chronic immune-mediated systemic disease [1] that affects genetically predisposed individuals, triggered by the ingestion of gluten. The only effective treatment is lifelong adherence to a strict gluten-free diet (GFD), which is crucial for managing symptoms and preventing long-term complications [2]. Despite its prevalence, affecting approximately 1% of the global population, awareness and diagnosis rates remain low, with only 36% of those affected in the UK being diagnosed [2]. This underdiagnosis is concerning, as untreated CD can lead to severe complications such as osteoporosis, malabsorption, and an increased risk of small bowel malignancies [3]. Recent findings highlight that the prevalence of undiagnosed CD from histology-based screening surveys in Europe ranges from 0.10% to 3.03% (median = 0.70%) and has shown a significantly increasing annual trend [4]. Prevalence since 2000 was highest in northern Europe (1.60%), compared to eastern (0.98%), southern (0.69%), and western Europe (0.60%) [4]. Diagnosed CD incidence has reached rates as high as 50 per 100,000 person-years in regions like Scandinavia, Finland, and Spain, with larger increases observed in older age groups compared to infants and young children [4]. This underscores the need for improved screening and awareness strategies to address underdiagnosis and its associated health risks.

Public awareness and understanding of CD are often limited. For example, a study conducted in Türkiye revealed that 43.9% of the surveyed population had never heard of the disease, with significant misconceptions regarding its nature and dietary management [5]. Similarly, findings from Saudi Arabia indicate that many individuals associate CD with wheat allergy, reflecting a gap in understanding its autoimmune etiology [6]. Moreover, the lack of societal sensitivity towards the challenges faced by individuals with CD, such as dining out and social activities, highlights the importance of raising public awareness to improve the quality of life for those affected [7]. Celiac disease is associated with various extraintestinal manifestations, including thyroid disorders, where vitamin D and iron play significant roles, as highlighted by Starchl et al. (2021) [8].

Patient education and healthcare provider training are essential to bridge these knowledge gaps. Studies have demonstrated that enhanced nutritional education programs, including interactive workshops and social media campaigns, can significantly improve adherence to a GFD and reduce the psychosocial burden of CD [9,10]. However, many healthcare providers report insufficient time for dietary follow-ups, and patients frequently rely on unreliable online sources for information [9].

This descriptive study aims to assess patient awareness, knowledge, and self-management of celiac disease while identifying gaps in understanding and care. The study seeks to highlight barriers to effective management and inform strategies to improve patient education, health outcomes, and quality of life.

## 2. Methodology

### 2.1. Survey Design

This study utilized a structured, anonymous online survey developed as part of the CD SKILLS project, funded by the European Union Regional Development Fund—Interreg Central Europe. The survey was designed to evaluate patient awareness, knowledge, and needs related to celiac disease (CD). The questionnaire was available in multiple languages and adhered to a standardized format to ensure consistency across regions. In this article, we present the results based on the answers of Bulgarian patients alone.

### 2.2. Participants

The survey targeted caregivers of children with celiac disease in Bulgaria, gathering insights into their experiences and perceptions regarding their children’s diagnosis, dietary management, and overall care. A total of 191 respondents participated in the survey, comprising both patients and caregivers. The inclusion criteria were as follows:

- A confirmed diagnosis of celiac disease for the respondent or their child.
- Voluntary participation.

### 2.3. Survey Structure

The questionnaire was divided into two primary sections as follows:

1. Knowledge assessment: Questions designed to test the participants’ understanding of celiac disease, its symptoms, complications, dietary management, and diagnostic procedures.
2. Patient experience and satisfaction: Questions assessing the respondents’ experience with medical and dietary support, availability of gluten-free products, and the overall healthcare system.

### 2.4. Key Themes Addressed

The survey covered the following key themes:

- Demographics: Gender, role (patient or caregiver), and familial adherence to a gluten-free diet.
- Knowledge of celiac disease: Including questions on disease definition, epidemiology, clinical presentation, complications, and high-risk groups.
- Dietary management: Understanding of gluten-free diets, food labeling, and adherence challenges.
- Healthcare support: Experience with diagnostic processes, medical care, and patient organization support.

### 2.5. Data Collection

The survey was distributed electronically, and responses were collected via the SurveyMonkey platform. Respondents were invited to participate through patient organizations and healthcare networks. The data collection period lasted four weeks, ensuring sufficient time for engagement.

### 2.6. Statistics

Descriptive statistics were employed to summarize the demographic characteristics, awareness, and diagnostic practices of the respondents. Categorical variables, including participant sex, caregiver roles, and diagnostic practices, were expressed as frequencies (n) and percentages (%). To assess the fit of the observed categorical data to the expected distributions, chi-square goodness of fit tests were conducted. For these tests, we assumed that the expected distributions were equal across all categories—meaning that, under the null hypothesis, each response option was anticipated to receive an equal number of responses. This neutral benchmark allows us to determine whether deviations from a uniform distribution are statistically significant, which may indicate underlying patterns or biases in patient awareness and diagnostic practices. Continuous variables, such as the time to diagnosis and diagnostic delays, were presented as the means and standard deviations (SD). All statistical analyses were conducted using Jamovi software v.2.1.2.

## 3. Results

### 3.1. Demographics and Diagnostic Knowledge of Celiac Disease

The results highlight critical trends and areas requiring targeted intervention. Female respondents, predominantly mothers, constituted 89% of participants. While 94.1% of respondents correctly identified celiac disease as a lifelong condition, only 26.7% accurately described it as a systemic immune-mediated disorder. Furthermore, awareness of the disease’s prevalence was low, with just 16.6% recognizing it as affecting 1 in 50–200 individuals (Table 1).

In terms of symptom awareness, the majority recognized classic gastrointestinal symptoms, and fewer than half (42.5%) were aware of potential asymptomatic manifestations. There was a high awareness of risk factors like family history (84.5%) and type 1 diabetes (45.9%) among respondents. However, less than half of participants recognized thyroid disease (38.7%) as one of the risk factors. Diagnostic awareness was strong regarding antibody testing (80.3%) and small intestine biopsy (88.8%)—the cornerstone of celiac disease diagnosis. However, there were misconceptions about the utility of abdominal ultrasound (34.3%) and full blood count (25.8%).

### 3.2. Dietary Practices in Celiac Disease

The results reveal significant insights into the awareness and practices related to celiac disease, underscoring key gaps and areas for improvement in public health education and patient support (Table 2).

Dietary adherence remains a cornerstone of Celiac disease management, with all respondents correctly identifying the lifelong need for a strict gluten-free diet (Table 2). 

Most households (69.7%) maintain strictly gluten-free environments, yet a notable portion (29.1%) operate in mixed settings with potential cross-contamination risks. Outside the home, the majority (83.6%) rely on bringing their own food, reflecting their limited trust in dining establishments and the availability of gluten-free certified options. Misunderstandings around grains containing gluten, such as buckwheat (35.47%), further highlight the need for targeted educational initiatives to address knowledge gaps (Table 2). 

### 3.3. Celiac Disease Diagnosis and Management Insights

Celiac disease diagnoses based on clinical symptoms and signs were reported by 95.0% of respondents, whereas only 4.1% were attributed to risk group screenings (Table 3). The mean time taken to visit a healthcare provider after symptom onset was 4.5 months (SD 5.7). The average time from the first consultation to the confirmation of diagnosis was 8.1 months (SD 15.6), with 38.8% of respondents unable to recall this period.

Interestingly, while 40.3% of diagnoses were confirmed by intestinal biopsy, 59.7% were made without it, aligning with the contemporary shift toward a non-biopsy diagnostic approach in selected cases. Among reasons for not performing an endoscopy, the most frequently cited was the physician deeming it unnecessary (57.83%). Pediatric gastroenterologists played a central role in confirming diagnoses, accounting for 76.0% of cases, followed by general gastroenterologists (19.4%).

### 3.4. Key Practices and Perceptions in Celiac Disease Management and Follow-Up

The results provide a comprehensive overview of the current practices and perceptions in managing celiac disease among respondents (Table 4). Regular follow-up practices show good adherence, with 45.1% of respondents visiting their physician within six months of diagnosis and 59.2% maintaining biannual follow-ups post-disease control. Misconceptions persist about the need for repeated biopsies, with 16.2% being uncertain about their necessity. Interestingly, 59.7% of diagnoses were made without a biopsy. Additionally, about 14.8% of respondents indicated a lack of awareness of complications, suggesting that a subset of patients may not fully comprehend the seriousness of the disease.

## 4. Discussion

The findings of this study provide valuable insights into the current state of awareness, diagnosis, and management of celiac disease in Bulgaria. While there is a growing acknowledgment of celiac disease as a lifelong systemic disease [1], our results reveal significant misconceptions, delays in diagnosis, and challenges in management that need to be addressed.

### 4.1. Awareness and Knowledge

Despite 94% of respondents correctly identifying celiac disease as a lifelong condition, only 26.7% accurately recognized it as a systemic immune-mediated disorder. This gap in understanding, along with the low awareness of the disease’s prevalence (16.6%), reflects findings from studies like that of Araya et al. (2014) [11], which highlights global underdiagnosis and poor knowledge of celiac disease. Such knowledge gaps may delay diagnosis and hinder early intervention efforts [12].

The study also revealed a concerning lack of awareness regarding the potential complications of untreated celiac disease. While 44.9% recognized osteoporosis, 40.3% identified intestinal lymphoma, and only 30.7% acknowledged infertility as possible outcomes. These findings are consistent with those of Ainsworth and Soon (2022) [13], who also reported limited patient education, even following a celiac diagnosis. This underscores the need for enhanced education on the long-term health risks associated with the disease. About 14.7% of respondents indicated a lack of awareness of any complications at all, suggesting that a subset of patients may not fully comprehend the seriousness of the disease. This suggests that while the necessity of the GFD is understood, the long-term health consequences of non-compliance may be underestimated. The GFD, while essential for managing celiac disease, also has broader applications and implications, as discussed by Aljada et al. (2021) [14].

The awareness of risk factors was higher for family history (84.5%) and type 1 diabetes (45.9%), which aligns with the findings of Farrier et al. (2024) [15]. However, only 38.7% recognized thyroid disease as a risk factor, suggesting that broader autoimmune associations are not sufficiently understood. This gap in knowledge emphasizes the importance of educating both patients and healthcare providers about the systemic nature of celiac disease to promote earlier detection and diagnosis. Children with celiac disease often face unique nutritional challenges, particularly when managing comorbid conditions like type 1 diabetes [16,17]. Our study underscores the need for comprehensive dietary education and support to ensure optimal nutritional status in these children. According to Ludvigsson et al. (2015) [18], enhancing patient education regarding the risks and complications associated with celiac disease is crucial for improving patient outcomes.

In terms of symptom awareness, while 90% of respondents correctly identified the classic gastrointestinal symptoms associated with celiac disease, fewer than half (42.7%) recognized that the condition can also present with asymptomatic manifestations. This gap in awareness underscores the need for comprehensive education that addresses the full spectrum of symptoms, particularly those that are not immediately linked to obvious gastrointestinal issues. Additionally, while diagnostic awareness was relatively strong, with 80.3% of participants understanding the importance of antibody testing and 88.8% recognizing the role of small intestine biopsy in confirming celiac disease, misconceptions about the diagnostic utility of abdominal ultrasound (34.3%) and full blood count (25.8%) highlight the necessity for clearer communication regarding the diagnostic process.

These findings emphasize the urgent need for comprehensive public health campaigns and targeted educational initiatives to enhance the understanding of celiac disease and its diagnostic procedures [19]. In addition to improving knowledge, addressing the availability and affordability of gluten-free resources is critical in ensuring equitable access for all patients. As previous research highlights, emotional and social support for celiac patients is equally essential, as many individuals report feeling isolated and misunderstood by family and friends (Taşkin and Savlak, 2021) [5]. Moreover, a recent study by Falcomer et al. (2024) [19] underscores how public policies targeting celiac disease, particularly those focusing on gluten-free meals and certification, must be more comprehensive to significantly improve the health-related quality of life (HRQoL) of individuals with celiac disease. Bridging these gaps, including the implementation of supportive public policies and improved patient education, is crucial not only for enhancing the management of celiac disease but also for improving the overall well-being and quality of life for those affected by the condition.

### 4.2. Delays in Diagnosis

The diagnostic delays observed in this study are concerning, with an average of 4.5 months between symptom onset and seeking medical care, and 8.1 months from the initial consultation to diagnosis confirmation. Alarmingly, 38.8% of respondents were unable to recall the time frame of their diagnostic journey, indicating a lack of awareness of the diagnostic process. Similar delays have been documented in earlier studies, such as that by Taylor et al. (2013) [20], who found that patients often consult multiple healthcare providers before receiving an accurate diagnosis. Riznik et al. (2021) [21] also identified significant knowledge gaps among both healthcare professionals and celiac disease patients, with an average knowledge score of just 50.9% for healthcare providers and 56.4% for patients, highlighting a broader issue with awareness. Research findings emphasize the need to enhance primary care providers’ awareness of celiac disease and improve patient education to reduce diagnostic delays. Such efforts could lead to faster and more accurate diagnoses, as well as improved patient adherence to treatment.

Interestingly, 59.7% of diagnoses in our study were made without intestinal biopsy, reflecting a growing reliance on the no-biopsy approach in select cases, especially among pediatric populations with strongly positive serology [11]. This shift aligns with the ESPGHAN and American College of Gastroenterology guidelines but also underscores the need for clear communication with patients about the diagnostic process to build trust in these evolving practices [1,22].

### 4.3. Challenges in Management

Despite the critical role of a gluten-free diet in managing celiac disease, only 32% of families in our study adhered strictly to gluten-free diets across all meals. This finding is consistent with Swift and Woodward’s (2018) [23] observation that dietary adherence rates range widely, from 42% to 91%, due to barriers such as cost, availability, and social stigma. In our study, misconceptions about grains containing gluten, such as oats (51.2%) and buckwheat (35.5%), further illustrate the need for targeted dietary education. While the gluten-free diet remains the cornerstone of celiac disease management, research is exploring other therapeutic strategies, as outlined by Mazzola et al. (2024) [24]. Our study highlights the importance of improving dietary adherence and awareness, which will be crucial as new treatments emerge.

The psychosocial impact of celiac disease is also evident, with 83.6% of respondents opting to bring their own food when dining outside due to limited trust in restaurant options. This finding is supported by Rose and Howard (2014) [25], who reported that celiac patients often experience social isolation and anxiety due to their dietary restrictions. Establishing stronger public awareness campaigns and fostering supportive communities can help alleviate these burdens, as suggested by the grounded theory study by Ainsworth and Soon (2022) [13], which highlighted the benefits of coeliac community formation in enhancing social integration.

### 4.4. Educational and Healthcare Implications

The low involvement of primary care physicians in confirming celiac disease diagnoses (0.8%) observed in our study highlights the need for improved training and resources at the primary care level. The current guidelines suggest that while GPs should conduct primary screening, the final diagnosis should be made by specialists, with referrals for positive test results. Additionally, Mc Elwenspoek et al. (2022) [26] emphasized the importance of an ‘active search’ approach, which involves screening high-risk groups and first-degree relatives to enhance detection rates.

Additionally, the low awareness of complications and poor adherence to dietary guidelines call for enhanced patient education efforts. As Lee et al. (2022) [27] noted, dietary counseling by a specialist dietitian should be a cornerstone of celiac disease management, addressing not only what to avoid but also how to navigate social, cultural, and emotional challenges.

### 4.5. Limitations

This study is based on patient recall, which can introduce potential inaccuracies, especially when reporting diagnostic delays. Although patient recall provides valuable insights into the patient experience, it may not be as accurate as data derived from health records. Additionally, male caregivers were significantly underrepresented, comprising only 11% of the sample. This highlights the need for more targeted efforts to engage and educate male family members and other caregivers, ensuring that they are equally involved in the management and understanding of celiac disease. Our study’s categorization of certain diagnostic tests, such as HLA testing, may not fully capture the nuances of real-world diagnostic practices. According to the ESPGHAN guidelines, HLA testing is not required for diagnosis in patients with positive serology and biopsy or high serum TGA-IgA levels with EMA positivity. However, it can provide supportive evidence in borderline cases. Another limitation is that our study’s sample is predominantly composed of caregivers, particularly parents of children with celiac disease, which may limit the generalizability of our findings. While this focus provides valuable insights into the challenges faced by families managing pediatric celiac disease, it may not fully capture the knowledge and experiences of adult patients with celiac disease. One more notable limitation of our study is the lack of detailed data on anti-endomysial antibody (EMA) testing, which is a fundamental component of the ESPGHAN criteria for the biopsy-free diagnosis of celiac disease. As such, we cannot confirm full adherence to the guideline-defined diagnostic algorithm in the subset of patients diagnosed without intestinal biopsy. This limits the precision with which we can evaluate diagnostic practices against international standards.

### 4.6. Future Directions

Future research should explore the long-term outcomes of patients managed under the no-biopsy approach, as well as the effectiveness of integrated care models that combine dietary counseling with psychosocial support. Longitudinal studies assessing the impact of educational interventions on awareness, diagnostic timelines, and adherence rates could provide valuable insights into improving care pathways for celiac patients.

## 5. Conclusions

This study highlights significant gaps in the awareness, diagnosis, and management of celiac disease. Addressing these challenges requires a multifaceted approach that includes public health campaigns, enhanced training for healthcare professionals, and accessible dietary support for patients. By implementing evidence-based strategies and leveraging insights from international guidelines, healthcare systems can improve outcomes and the quality of life for individuals living with this chronic condition.

## Figures and Tables

**Table 1:** Key findings on the demographics and diagnostic knowledge of celiac disease among survey respondents.

Question	Answer Choices	Responses (n)	Percent (%)
**Gender** (X^2^ = 116.24, *df* = 1 *p* = 0.0001)	Female	170	89.0%
	Male	21	11%
**CD respondent** (X^2^ = 251.38, *df* = 6 *p* = 0.0001)	Patient	38	20%
	Mother	123	64.4%
	Father	9	4.7%
	Caregiver (guardian)	14	7.3%
	Both patient and caregiver	7	3.6%
**Number of children in the family with CD** (X^2^ = 121.68, *df* = 1 *p* = 0.0001)	1	144	94.7%
	2	8	5.3%
**Time since diagnosis** (X^2^ = 105.79, *df* = 3 *p* = 0.0001)	<6 months	9	4.7%
	>6 months–<1 year	20	10.6%
	>1 year–<5 years	62	32.8%
	5 years or more	98	51.9%
**CD description** (X^2^ = 138.91, *df* = 4 *p* = 0.0001)	Autoimmune disease affecting only the small intestine	93	49.7%
	Systemic immune-mediated disease in predisposed people	50	26.7%
	Allergic disease in atopic families	Not selected as response	
	Food intolerance affecting small intestine	16	8.6%
	Enzyme deficiency affecting gluten breakdown	28	15%
**CD lifespan and treatment** (X^2^ = 476.85, *df* = 3 *p* = 0.0001)	Lifelong	176	94.1%
	Curable	1	0.5%
	Sometimes curable	7	3.8%
	I don’t know	3	1.6%
**Age of onset** (X^2^ = 529.56, *df* = 3 *p* = 0.0001)	Only in children	1	0.5%
	At any age	183	97.9%
	Only in adults	Not selected as response	
	I don’t know	3	1.6%
**CD prevalence** (X^2^ = 56.34, *df* = 4 *p* = 0.0001)	1 per 1000 or more	73	39.0%
	1 per 500–1000	29	15.5%
	1 per 200–500	11	5.9%
	1 per 50–200	31	16.6%
	I don’t know	43	23%
**CD clinical presentation** (X^2^ = 166.41, *df* = 5 *p* = 0.0001) (multiple selections possible)	Always with GI symptoms	74	39.8%
	Can present with GI symptoms	102	54.8%
	Never with GI symptoms	1	0.5%
	Can present without GI symptoms	66	35.5%
	Can be asymptomatic	79	42.5%
	I don’t know	2	1.1%
**High-risk groups for celiac disease** (X^2^ = 235.60, *df* = 6 *p* = 0.0001) (multiple selections possible)	Thyroid disease	70	38.7%
	Type 1 diabetes	83	45.9%
	First-degree relatives	153	84.5%
	Eczema (chronic dermatitis)	42	23.2%
	Asthma	10	5.5%
	Other (e.g., autoimmune diseases, vitiligo)	11	6.1%
**Specific diagnostic tests for celiac disease** (X^2^ = 283.96, *df* = 6 *p* = 0.0001) (multiple selections possible)	Antibody tests	143	80.3%
	Small intestine biopsy	158	88.8%
	Genetic tests	134	75.3%
	Abdominal ultrasound	61	34.3%
	Full blood count	46	25.8%
	Colon biopsy	27	15.2%
	I don’t know	3	1.7%

Note: Correct answers are indicated in yellow, where appropriate.

**Table 2:** Dietary practices in celiac disease.

Question	Answer Choices	Responses (n)	Response (%)
**Recommended gluten-free diet** (X^2^ = 176, *df* = 1 *p* = 0.0001)	Lifelong strict gluten-free diet	176	100%
	Other options (e.g., reduced gluten intake)	Not selected as response	
**Family members following gluten-free at home** (X^2^ = 67.28, *df* = 1 *p* = 0.0001)	Yes, all meals	31	31%
	No	68	69%
**Knowledge of gluten containing grains** (X^2^ = 305.87, *df* = 8 *p* = 0.0001) (multiple selections possible)	Wheat	170	98.8%
	Barley	161	93.6%
	Rye	166	96.5%
	Oats	88	51.7%
	Spelt	166	96.5%
	Buckwheat	61	35.5%
	Corn	8	4.7%
	Millet	21	12.2%
	Kamut	161	93.6%
**Preparation of gluten-free food at home** (X^2^ = 34.48, *df* = 2 *p* = 0.0001)	Strictly gluten-free environment	115	69.7%
	Mixed environment (gluten-free and non-gluten-free)	48	29.1%
	Other	2	1.2%
**Eating outside the home** (X^2^ = 213.17, *df* = 4 *p* = 0.0001) (multiple selections possible)	Bring your own food	138	83.6%
	Eat anywhere, providing instructions to the chef	34	20.6%
	Eat at gluten-free certified restaurants	22	13.3%
	Eat at restaurants with gluten-free menu options	36	21.8%
	Other	13	7.9%

Note: Correct answers are indicated in yellow, where appropriate.

**Table 3:** Celiac disease diagnosis and management insights.

Question	Answer Option	Responses (n)	Responses (%)
**Basis for CD diagnosis** (X^2^ = 102.81, *df* = 1 *p* = 0.0001)	Symptoms/signs	117	95.9%
	Risk group screening	5	4.1%
**Time to doctor visit after symptoms** (X^2^ = 17.12, *df* = 1 *p* = 0.0001)	Don’t remember	41/129	31.8%
Mean (SD)	4.5 (5.7) Months	88	68.2%
**Time to final diagnosis** (X^2^ = 6.52, *df* = 1 *p* = 0.0106)	Don’t remember	50/129	38.8%
Mean (SD)	8.1 (15.6) Months	79	61.2%
**Intestinal biopsy confirmation** (X^2^ = 2.24, *df* = 1 *p* = 0.13)	Yes	56	40.3%
	No	83	59.7%
**Reasons for not performing endoscopy** (X^2^ = 102.81, *df* = 1 *p* = 0.0001)	Risk due to another medical condition	11	13.25%
	Not available	7	8.43%
	Deemed unnecessary by the physician	48	57.83%
	Financial reasons	1	1.2%
	Patient/guardian declined	12	14.46%
	Don’t know	4	4.82%
**Confirming CD diagnosis** (X^2^ = 347.42, *df* = 5 *p* = 0.0001)	Primary care physician (e.g., family doctor, pediatrician)	1	0.8%
	Pediatric gastroenterologist	98	76.0%
	Gastroenterologist	25	19.3%
	Practitioner of alternative medicine	Not selected as response	
	Self-diagnosed	2	1.6%
	Other	3	2.3%

**Table 4:** Key practices and perceptions in celiac disease management and follow-up.

Question	Answer Option	Responses (n)	Responses (%)
**First follow-up visit post-diagnosis** (X^2^ = 51.86, *df* = 3 *p* = 0.0001)	1 month	38	23.2%
	3 months	43	26.2%
	6 months	74	45.1%
	Other	9	5.5%
**Frequency of follow-up visits** (X^2^ = 81.76, *df* = 2 *p* = 0.0001)	Every 6 months	93	59.2%
	Annually	62	39.5%
	Every 2 years	2	1.3%
**Recommendation after symptom improvement** (X^2^ = 310.99, *df* = 3 *p* = 0.0001)	Continue gluten-free diet	137	85.1%
	Reintroduce gluten after control confirmation	2	1.2%
	Gluten challenge and retesting	10	6.2%
	Don’t know	12	7.5%
**Need for repeated intestinal biopsies** (X^2^ = 162.25, *df* = 2 *p* = 0.0001)	Yes	6	3.7%
	No	129	80.1%
	Don’t know	26	16.2%
**Complications of untreated CD** (X^2^ = 208.8, *df* = 9 *p* = 0.0001) (multiple selections possible)	Anemia	114	64.8%
	Osteoporosis	79	44.9%
	Intestinal lymphoma	71	40.3%
	Infertility	54	30.7%
	Celiac crisis	84	47.7%
	Chronic liver disease	35	19.9%
	Chronic inflammatory bowel diseases	105	59.7%
	Epilepsy	9	5.1%
	Chronic kidney disease	11	6.3%
	I don’t know	26	14.8%

Note: Correct answers are indicated in yellow, where appropriate.

## Data Availability

The original contributions presented in this study are included in the article. Further inquiries can be directed to the corresponding author.

## Abbreviations

CD	Celiac Disease
GFD	Gluten-Free Diet
GI	Gastrointestinal
HRQoL	Health-Related Quality of Life
SD	Standard Deviation
X^2^	Chi-Square Test
